# Characterization of a novel member of the family *Caulimoviridae* infecting *Dioscorea nummularia* in the Pacific, which may represent a new genus of dsDNA plant viruses

**DOI:** 10.1371/journal.pone.0203038

**Published:** 2018-09-12

**Authors:** Amit C. Sukal, Dawit B. Kidanemariam, James L. Dale, Robert M. Harding, Anthony P. James

**Affiliations:** 1 Centre for Tropical Crops and Biocommodities, Queensland University of Technology, Brisbane, Queensland, Australia; 2 Centre for Pacific Crops and Trees, Pacific Community, Suva, Fiji; Washington State University, UNITED STATES

## Abstract

We have characterized the complete genome of a novel circular double-stranded DNA virus, tentatively named Dioscorea nummularia-associated virus (DNUaV), infecting *Dioscorea nummularia* originating from Samoa. The genome of DNUaV comprised 8139 bp and contained four putative open reading frames (ORFs). ORFs 1 and 2 had no identifiable conserved domains, while ORF 3 had conserved motifs typical of viruses within the family *Caulimoviridae* including coat protein, movement protein, aspartic protease, reverse transcriptase and ribonuclease H. A transactivator domain, similar to that present in members of several caulimoviridae genera, was also identified in the putative ORF 4. The genome size, organization, and presence of conserved amino acid domains are similar to other viruses in the family *Caulimoviridae*. However, based on nucleotide sequence similarity and phylogenetic analysis, DNUaV appears to be a distinct novel member of the family and may represent a new genus.

## Introduction

Yams (*Dioscorea* spp.) are ranked as the fourth most important root crop by production after potato, cassava and sweet potato. They provide a staple food source for millions of people in Africa, the Caribbean, South America, Asia and the Pacific [1] while wild yams provide a valuable food source in times of famine. Yam production is highest in West Africa, which accounts for 95% of the world’s total production [2]. Although most of the production occurs in the African region, predominated by *Dioscorea rotundata-cayenensis*, yam is of importance in South Pacific countries where *D*. *alata* and *D*. *esculenta* are the dominant species [3] with some scattered cultivation of *D*. *rotundata*, *D*. *bulbifera*, *D*. *nummularia*, *D*. *transversa* and *D*. *trifida* throughout the region.

Yam cultivation and improvement in the Pacific faces many agronomical challenges including yield losses due to pests and diseases [4,5]. To help address these issues, as well as improve food security and facilitate commercial agricultural opportunities in the Pacific region, access to germplasm from the Pacific and other regions (such as Africa) is needed for possible exploitation. An important collection of Pacific yam germplasm is conserved in tissue culture at the Centre for Pacific Crops and Trees (CePaCT) of the Pacific Community (SPC), Suva, Fiji. This collection, together with yam germplasm from the International Institute of Tropical Agriculture (IITA) in West Africa, could hold the key to addressing the problems faced with yam cultivation in the Pacific. However, like many other vegetatively propagated crops such as sugarcane, banana, cassava, aroids and sweet potato, yams are prone to virus infection and accumulation. Therefore, the identification of viruses infecting the crop and the development of reliable diagnostic tests is critical to facilitate the safe exchange and utilization of yam germplasm.

Viruses belonging to the families *Alphaflexiviridae* (genus *Potexvirus*), *Betaflexiviridae* (genus *Carlavirus*), *Bromoviridae* (genus *Cucumovirus*), *Caulimoviridae* (genus *Badnavirus*), *Potyviridae* (genus *Macluravirus* and *Potyvirus*), *Secoviridae* (genus *Comovirus* and *Fabavirus*) and *Tombusviridae* (genus *Aureusvirus*) are known to infect yams [6,7]. Of these, viruses belonging to the family *Caulimoviridae* remain the least studied and the most difficult to diagnose due to their significant genetic variability and, in some cases, the presence of integrated viral sequences in the host genome [8–10].

The family *Caulimoviridae* consists of eight genera of reverse transcribing, double-stranded DNA (dsDNA)-containing plant viruses, which are primarily distinguished from each other based on particle morphology and genome organization [11,12]. Six of the genera, namely *Caulimovirus*, *Cavemovirus*, *Petuvirus*, *Rosadnavirus*, *Soymovirus* and *Solendovirus* have isometric virions that are 52 nm in diameter, while two genera, *Badnavirus* and *Tungrovirus*, have bacilliform virions with dimensions of 30 x 130 to 150 nm [11,13]. All family members have a genome size between 7.2 to 9.2 kb with the coding capacity on the plus-strand. To date, only species belonging to the genus *Badnavirus* have been identified from yams, namely Dioscorea bacilliform alata virus (DBALV), DBALV2, Dioscorea bacilliform esculenta virus (DBESV), Dioscorea bacilliform rotundata virus 1 (DBRTV1), DBRTV2, DBRTV3, Dioscorea bacilliform sansibarensis virus (DBSNV) and Dioscorea bacilliform trifida virus (DBTRV) [9,14–18]. In addition to these full-length viral sequences, a large number of partial reverse transcriptase (RT)-ribonuclease H (RNase H) sequences which cluster within numerous different monophyletic groups have also been PCR-amplified from yam germplasm [3,9,19–22]. While the majority of these groups cluster within the genus *Badnavirus*, several groups do not cluster with any recognized genera within the family *Caulimoviridae* [3,21]. Whether these sequences are derived from episomal or integrated viral sequences or from another source such as retrotransposons is unknown since they were generated by PCR.

In 2014, a project was initiated to characterize the diversity of badnaviruses infecting yams in the Pacific region. In this paper, we report the identification of a putative new member of the family *Caulimoviridae* from yam, tentatively named Dioscorea nummularia-associated virus (DNUaV). The genome properties and organization of DNUaV are described and its relationship to other members of the family *Caulimoviridae* is discussed.

## Materials and methods

### Plant material and nucleic acid extraction

CePaCT maintains an *in vitro* collection of yams (278 accessions) which is comprised of seven different species: *D*. *alata* (n = 193), *D*. *rotundata* (n = 32), *D*. *esculenta* (n = 41), *D*. *bulbifera* (n = 8), *D*. *nummularia* (n = 2), *D*. *transversa* (n = 1) and *D*. *trifida* (n = 1) originating from Africa (obtained from IITA, Ibadan, Nigeria), Papua New Guinea (PNG), Vanuatu, New Caledonia, Federated States of Micronesia (FSM), Samoa and Tonga. Following screenhouse acclimatization for three months leaf samples from 173 plants representative of the collection were used in this study. Total nucleic acid (TNA) was extracted using a CTAB protocol [23] from approximately 100 mg of fresh leaf tissue. The purified TNA was treated with RNase A (1 μg/μl) and the concentration adjusted to 500 ng/μl with sterile nuclease-free water.

### RCA and sequencing

RCA was done essentially as described previously [24]. Briefly, 1 μl of TNA extract was used as template in RCA using the TempliPhi^™^ 100 Amplification Kit (GE Healthcare, UK) with the addition of 1 μl of 10 mM 3’-exonuclease-protected degenerate badnavirus primers BadnaFP/RP [25] to bias amplification towards badnavirus DNA.

RCA products were independently digested with *Eco*RI, *Kpn*I, *Sph*I and *Stu*I restriction endonucleases which were selected following *in silico* restriction analysis of published yam badnavirus genome sequences, or based on experimental experience, to generate useful restriction profiles. Digested RCA products were electrophoresed through 1% agarose gels at 100 V for 1 h. Restriction fragments of approximately full-length genome size (7–8 kb) were excised and ligated into appropriately digested and de-phosphorylated pUC19. Plasmids were first screened via restriction analysis to ensure desired inserts were present, then subjected to Sanger sequencing using either universal M13 primers or BadnaFP/RP primers. The resulting sequences were used to query the National Centre for Biotechnology Information (NCBI) database (www.ncbi.nlm.nih.gov) with the BLASTn and BLASTx search functions. Where BLAST analysis yielded a match to viral sequences, primer walking using sequence-specific primers was used to generate full-length sequences in both directions.

To confirm the sequences spanning putative restriction sites, PCR was carried out using sequence-specific primers flanking the region. PCR mixes consisted of 10 μl of 2x GoTaq Green Master Mix (Promega, USA), 5 ρmol of each sequence-specific primer and 1 μl of DNA extract (diluted to ~50 ng/μl) in a final volume of 20 μl. PCR cycling conditions were as follows: initial denaturation at 94°C for 2 min followed by 35 cycles of 94°C for 20 s, 50°C for 30 s and 72°C for 2 min, with a final extension at 72°C for 10 min. Amplicons were cloned into pGEM^®^-T Easy (Promega, USA) and sequenced with primers M13F/R as described previously.

Putative full-length sequences were assembled using Geneious v11.0.5 [26]. SnapGene^®^ software (www.snapgene.com; GSL Biotech) and ORFfinder (https://www.ncbi.nlm.nih.gov/orffinder/) were used to predict putative ORFs on the plus-strand of the assembled full-length sequences. InterPro software was used to scan protein databases for conserved domains [27], while BLASTn and BLASTx were used to search for sequence homologies in GenBank.

### Sequence comparisons and phylogenetic analyses

Pairwise sequence comparison (PASC) was done using sequences corresponding to amino acid residues L_269_-R_672_ of the cauliflower mosaic virus (CaMV) polymerase (*pol*) gene. This region includes the conserved motifs of the RT- and RNase H-coding regions [28] and is currently used for the demarcation of species in the family *Caulimoviridae* [12]. Nucleotide or translated amino acid sequences were aligned using ClustalW alignment in MEGA7 [29].

Phylogenetic analyses were done using the nucleotide sequences of either the 529 bp RT/RNase H-coding region delineated by the BadnaFP/RP primer binding sites or the *pol* gene sequences described above. Sequences were aligned using ClustalW and phylogenetic trees were constructed using the maximum-likelihood method (Kimura-2-parameter model) in MEGA7 with 1000 bootstrap replication.

### Viral DNA detection

Specific primers DNUaV-ORF4-FP1 (5'-CCGGGTTGCCAGTACAGAAT-3') and DNUaV-ORF4-RP1 (5'-CGTGAAGCACCCAAACCTTG-3') were designed following sequence analysis to amplify a 450 bp region of the putative ORF 4 sequence. PCR was carried out using GoTaq Green essentially as described previously using 57°C as the annealing temperature. Amplicons were cloned and sequenced as described earlier.

## Results

### Identification of DNUaV

Of the 173 samples analyzed, none of which showed symptoms, 35 yielded restriction profiles indicative of the presence of badnaviruses. Restriction analysis of RCA products derived from two Samoan *D*. *nummularia* accessions (DN/WSM-01 and DN/WSM-02) using *Sph*I and *Stu*I, resulted in putative full-length products (~8 kb), while *Kpn*I gave no digest products and digestion using *Eco*RI resulted in a number of products smaller than 3.5 kb. These profiles were inconsistent with those expected for known yam-infecting badnaviruses based on analysis of full-length sequences available in GenBank. Therefore, the putative full-length *Sph*I digested fragments were cloned and sequenced. Sequences originating from the termini of the ~8 kb *Sph*I fragments from both samples showed no nucleotide similarity with published viral sequences. However, BLASTx analysis revealed that the putative amino acid sequence from one end of the cloned fragments had low (32%) similarity to the ORF 1 protein of the badnavirus, cacao yellow vein-banding virus (CYVBV), and 31% similarity to the ORF 1 protein of the tungrovirus, rice tungro bacilliform virus. Sequencing of the cloned fragments was subsequently carried out using the degenerate badnavirus primers BadnaFP/RP. Sequences were only obtained using primer BadnaFP, with BLASTn analysis revealing 73–75% identity with two partial RT/RNase H-coding sequences of a Dioscorea bacilliform virus derived from *D*. *nummularia* (GenBank accession numbers AM072692 and AM421696). Since the sequences of the two 8 kb-*Sph*I clones from isolates DN/WSM-01 and DN/WSM-02 showed 99% nucleotide similarity, the complete genomic sequence of only one isolate, DN/WSM-01, was determined. This sequence was obtained from three independent clones using primer walking, and the presence of the single *Sph*I restriction site was confirmed through additional PCR analysis and sequencing.

### Genome organization, sequence and phylogenetic analysis

The complete genomic sequence of the virus isolate derived from yam accession DN/WSM-01 was 8139 bp in length and was deposited in GenBank under the accession number MG944237. Consistent with the RFLP patterns observed, the genome contained 5 *Eco*RI sites, single *Sph*I and *Stu*I sites, and no *Kpn*I site.

**Fig 1:**
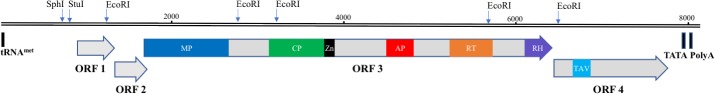
Schematic representation of the genome organization of Dioscorea nummularia-associated virus (DNUaV). Large arrows represent the putative ORFs. Conserved protein domains are shown: dark blue = movement protein (MP) domain corresponding to M_1_-E_327_ of CaMV ORF 2 protein; green = the putative coat protein (CP) domain corresponding to L_261_–N_429_ of the CaMV ORF 4 protein; black = zinc finger (Zn); red = pepsin-like aspartic protease (AP); orange = reverse transcriptase (RT); purple = RNase H (RH); light blue = transactivator (TAV) domain. The tRNA^met^ binding site (tRNA^met^) and regulatory sequences including TATA box (TATA) and polyadenylation signal (PolyA) are also shown. The relative position of restriction sites based on the complete genome sequence are shown above the genome.

The genome of isolate DN/WSM-01 contained four putative ORFs which comprised 450 nt (ORF 1), 384 nt (ORF 2), 4737 nt (ORF 3) and 1371 nt (ORF 4) (Fig 1). ORFs 1 and 2, and 2 and 3 overlapped, whereas ORFs 3 and 4 were separated by one nucleotide. Whereas ORFs 1 and 2 had overlapping stop/start codons (atga), the putative start codon of ORF 3 was located 47 nucleotides 5' of the ORF 2 stop codon (Fig 1). ORF 2 was in a -1 translational reading frame relative to ORF 1, while ORF 3 was in a +1 translational reading frame relative to ORF 2. The genome contained one large intergenic region (IR), between ORF 4 and ORF 1 which comprised 1247 nt and contained a putative tRNA^met^ binding site (5'-TGGTATCAGAGCAATGGT-3') with 88% nucleotide similarity to the plant tRNA^met^ consensus sequence (3'-ACCAUAGUCUCGGUCCAA-5'), which has been described as the priming site for reverse transcription [30]. This was designated as the origin of the circular genome, consistent with the convention used for other caulimoviridae members. A TATA-box (TATATAA_7944-7950_) and polyadenylation signal (AAAAAATAA_7981-7989_), analogous to the 35S promotor of CaMV, were also identified in the region 5' of the tRNA^met^ site.

**Fig 2:**
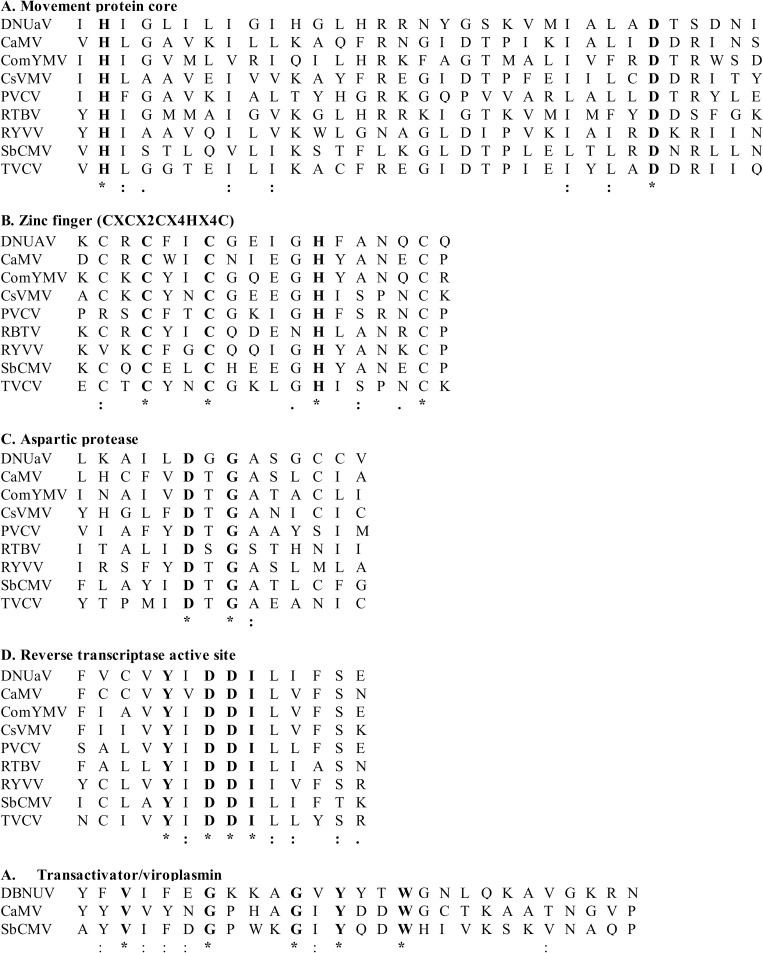
Amino acid sequence alignments of the conserved motifs in the proteins of the type member of each genus in the family *Caulimoviridae*. The type member for each genus within the family *Caulimoviridae* used for comparison is as follows: *Caulimovirus*—cauliflower mosaic virus (CaMV; V00141), *Badnavirus—*Commelina yellow mottle virus (ComYMV; X52938), *Cavemovirus—*cassava vein mosaic virus (CsVMV; U59751), *Petuvirus—*Petunia vein clearing virus (PVCV; U95208), *Tungrovirus—*rice tungro bacilliform virus (RTBV; NC001914), *Rosadnavirus—*rose yellow vein virus (RYVV; JX028536), *Soymovirus*—soybean chlorotic mottle virus (X15828), *Solendovirus*—tobacco vein clearing virus (TVCV; AF190123). Identical (asterisk/bold font), conserved (colon) and weakly conserved (dot) residues among the members of the family are indicated.

Analysis of the translated ORF sequences failed to identify any conserved domains in ORFs 1 and 2. In contrast, comparative sequence analysis of ORF 3 revealed several functional domains shared by all members of the family *Caulimoviridae* including aspartic protease (Ala_933_-Ile_1045_, IPR021109), zinc finger (Cys_703_-Cys_708_, IPR001878), RT (Lys_1187_-Ile_1348_, IPR000477) and RNase H domains (Ser_1469_-Ala_1574_, IPR002156). In addition, a conserved movement protein (MP) domain corresponding to M_1_-E_327_ of CaMV ORF 2 protein, and a coat protein (CP) domain corresponding to L_261_–N_429_ of the CaMV ORF 4 protein, were also identified. A transactivator (TAV) domain (Tyr_80_-Thr_122_, pfam01693), similar to that present in ORF 6 of caulimoviruses and soymoviruses, was identified in the putative ORF 4 sequence (Figs 1 and 2A–2E).

When the full-length genome sequence of isolate DN/WSM-01 was used for BLAST analysis with the search restricted to viruses (taxid:10239), the highest nucleotide identity (70%) was to a 263 bp and 186 bp region of the RT domain of two members of the genus *Badnavirus*, namely DBRTV2 (accession KX008579) and cacao swollen shoot virus (CSSV; accession KX592572.1), respectively. BLAST analysis of the putative protein sequences encoded by ORFs 1–4 of isolate DN/WSM-01 revealed highest similarity with the ORF 1 protein of CYVBV (32%), ORF 2 protein of taro bacilliform virus (32%), ORF 3 polyprotein of fig badnavirus 1 (41%), while ORF 4 had 26% similarity to amino acids 1443 to 1544 of Piper DNA virus 1.

**Fig 3:**
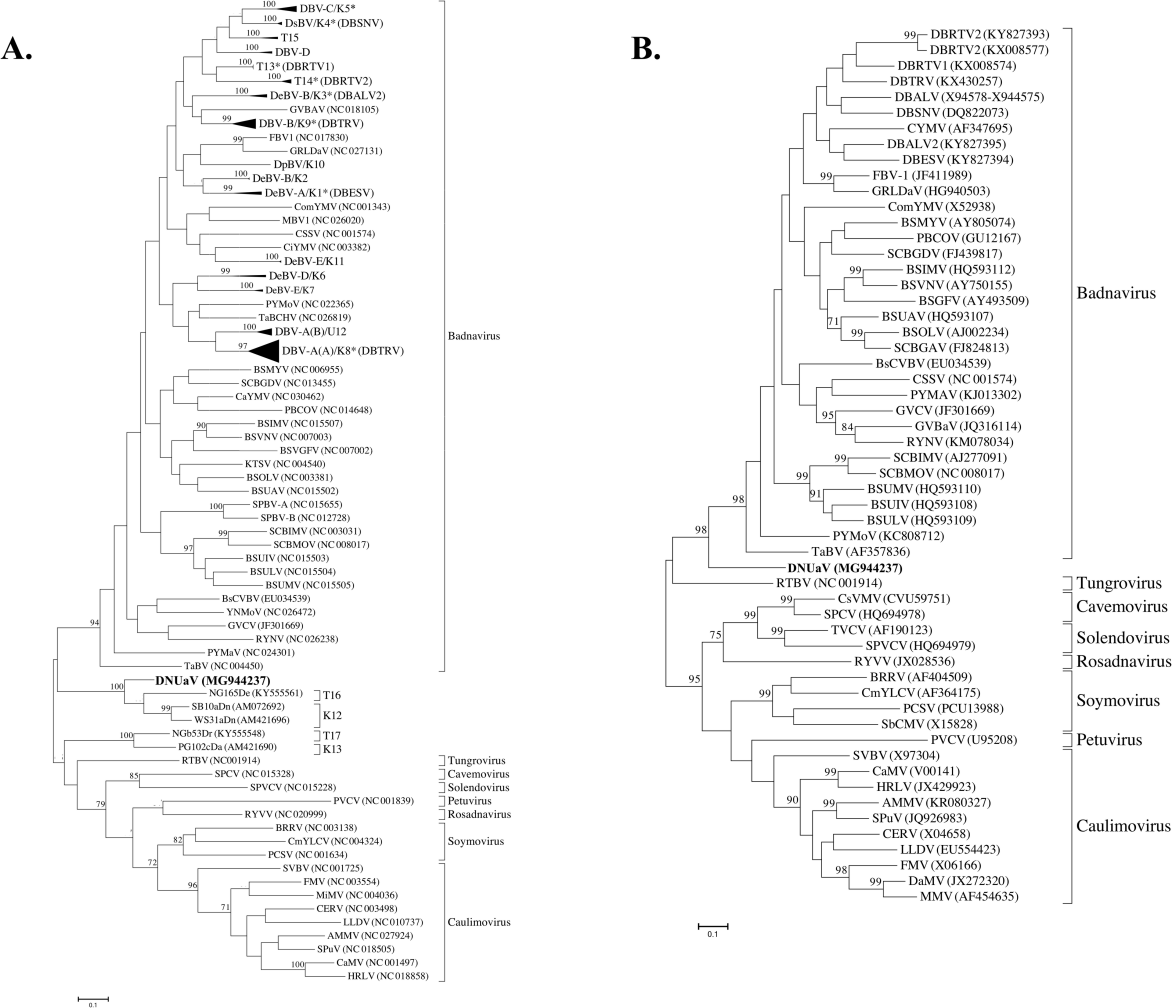
Phylogenetic analysis using the maximum-likelihood method following ClustalW alignment in MEGA7 (29) to infer evolutionary relationships of DNUaV. Bootstrap values (1,000 replicates) are shown above nodes when greater than 70%. (A) Phylogenetic tree constructed using sequences of the RT/RNase H-coding region delineated by the BadnaFP/RP primers (25). This analysis includes badnavirus RT/RNase H-coding sequences identified from yams [3,9,19–22], badnaviruses infecting other crops and the homologous region of other caulimoviridae members (See S1 Table and S2 Table for complete list of sequences used in the analysis). Subgroups representing published yam badnavirus sequences have been collapsed to improve the presentation of the tree, while the badnavirus groups that have representative full genome sequence available are marked with an asterisk (*); (B) Phylogenetic tree using *pol* gene nucleotide sequences of DNUaV and representative members of family *Caulimoviridae* (See S2 Table for list of sequences included in the analysis). The *pol* gene sequences are equivalent to amino acid residues L_269_-R_672_ from the translated ORF 5 nucleotide sequence of cauliflower mosaic virus (CaMV).

PASC using either nucleotide or translated amino acid sequences of the *pol* gene revealed an identity of 43 to 58% or 32 to 53%, respectively, between DNUaV and the type species for each genus in the family *Caulimoviridae* (Table 1). Phylogenetic analysis using partial RT/RNase H-coding sequences showed that DNUaV forms a distinct subgroup outside of the genus *Badnavirus*, together with several published sequences (GenBank accession numbers KY555561, AM072692 and AM421696) previously reported from yams (Fig 3A). A similar tree topology, with DNUaV clustering separately from recognized caulimoviridae genera, was obtained when *pol* nucleotide sequences from published full-length sequences were analyzed (Fig 3B).

**Table 1:** Mean pairwise nucleotide (above diagonal) and amino acid (below diagonal) similarity between the *pol* gene of DNUaV and the type members of the eight current genera within the family *Caulimoviridae* ^a^.

	DNUaV	CaMV	ComYMV	CsVMV	PVCV	RTBV	RYVV	SbCMV	TVCV
DNUaV		48	58	48	45	54	47	45	47
CaMV	41		46	46	51	47	48	48	47
ComYMV	53	36		43	42	49	45	42	43
CsVMV	36	36	32		45	48	54	45	64
PVCV	32	42	29	32		43	46	44	44
RTBV	45	36	40	33	30		46	43	48
RYVV	39	40	35	39	34	35		44	51
SbCMV	32	39	28	27	29	27	32		43
TVCV	38	37	32	48	30	36	37	28	

^a^Abbreviations for the type members of each genus are: *Caulimovirus—*cauliflower mosaic virus (CaMV; V00141), *Badnavirus—*Commelina yellow mottle virus (ComYMV; X52938), *Cavemovirus—*cassava vein mosaic virus (CsVMV; U59751), *Petuvirus—*Petunia vein clearing virus (PVCV; U95208), *Tungrovirus—*rice tungro bacilliform virus (RTBV; NC001914), *Rosadnavirus—*rose yellow vein virus (RYVV; JX028536), *Soymovirus*—soybean chlorotic mottle virus (X15828), *Solendovirus*—tobacco vein clearing virus (TVCV; AF190123) used in the analysis above.

### PCR screening for DNUaV

Using primers designed to amplify a 450 bp region of DNUaV ORF 4, the 173 samples used in this study were tested for DNUaV by PCR. The expected size amplicon was only generated from the Samoan *D*. *nummularia* samples, DN/WSM-01 and DN/WSM-02. Sequence analysis of the cloned PCR amplicons from the two samples revealed 99% similarity to each other and to the DNUaV ORF4 sequence generated using RCA.

## Discussion

In this study, we identified and characterized a novel DNA virus infecting *D*. *nummularia* which we have tentatively named Dioscorea nummularia-associated virus (DNUaV). Although the genome size and organization, and the presence of conserved amino acid domains of DNUaV, is typical of other viruses in the family *Caulimoviridae*, there are several molecular features of the virus that distinguish it from the current genera.

The ICTV uses several criteria to classify members of the family *Caulimoviridae*. The most common criterion for demarcation of species uses differences in the nucleotide sequence of the *pol* gene (AP/RT/RNase H-coding region) of more than 20%. Comparisons of the *pol* gene sequence of DNUaV with other *Caulimoviridae* showed the highest identity (76%) to a partial sequence of Dioscorea bacilliform virus isolate SB10a_Dn derived from *D*. *nummularia* [3]. Based on differences in the nucleotide sequence identity of more than 20%, DNUaV appears to be a novel virus in the family *Caulimoviridae*.

In addition to nucleotide sequence similarity, distinctions between genera within the family *Caulimoviridae* are also based on the type of host plant, particle morphology, genome organization and the presence and arrangement of conserved protein-coding motifs. DNUaV encodes four ORFs with the size of ORFs 1–3 consistent with both badnavirus and tungrovirus members, as are the arrangement of the characteristic MP, CP, Zn-finger binding domain and the AP-RT-RNase H-coding regions of ORF 3. The relative positions of ORF 1 and 2 are similar to those of badnaviruses, while ORFs 2 and 3 overlap each other by 47 nt which is also similar to the badnaviruses CSSV, gooseberry vein banding virus, Piper yellow mottle virus and sweet potato pakakuy virus [31–33]. However, unlike those badnaviruses with a fourth ORF which always overlaps with ORF3, ORF4 of DNUaV is separated from ORF 3 by a short intergenic region which is more similar to genome organization of RTBV, the sole member of the genus *Tungrovirus*. Further, the size of DNUaV ORF 4 is also similar to that of RTBV. Unlike RTBV, however, the DNUaV ORF 4 gene product contains a conserved translation transactivator domain, which is typical of ORF 6 of caulimoviruses and soymoviruses, and which is also present in ORF 4 of cavemoviruses and solendoviruses. However, unlike the DNUaV ORF 4 sequence, the ORF 4 sequences of both cavemoviruses and solendoviruses also includes the conserved coiled-coil motifs characteristic of the virion-associated protein. Clearly, determination of virion morphology and whether infected plants contain inclusion bodies typical of members of the genus *Caulimovirus* is required before the taxonomic status of DNUaV can be fully resolved. However, based on the sequence information presented, DNUaV appears to be a distinct, novel member of *Caulimoviridae*.

PASC carried out using *pol* gene sequences showed 45 to 58% nucleotide or 32 to 53% amino acid sequence identity between DNUaV and the type members of each genus within the family *Caulimoviridae* (Table 1). This level of nucleotide sequence identity is typical of that between the established genera within the family *Caulimoviridae*, which ranges from 42 to 64% (Table 1). Further, the level of amino acid sequence identity is similar to the range of 27 to 48% identity between the type members of each genus. Of the eight type members included in the analysis DNUaV shares the highest level of amino acid identity (53%) with ComYMV, the type member of the genus *Badnavirus* (Table 1), suggesting that DNUaV is most closely related to the badnaviruses. However, phylogenetic analyses using either partial RT/RNase H-coding sequences (Fig 3A) or *pol* gene sequences (Fig 3B), indicates that DNUaV is basal to, and distinct from, the badnaviruses, forming a distinct clade between the single member of the genus *Tungrovirus*, RTBV, and the genus *Badnavirus*. This suggests that DNUaV may belong in a new, distinct genus within the family *Caulimoviridae*.

Previous studies investigating the occurrence of badnaviruses in yams have reported large numbers of badnavirus partial RT/RNase H-coding (529 bp) sequences generated using the BadnaFP/RP primers [3,9,10,18–22]. Phylogenetic analyses of these sequences identified four distinct sequence groups, namely K12/K13 [3] and T16/T17 [21], which clustered into two monophyletic groups (K12/T16 and K13/T17) outside of the eight currently recognized genera within the family *Caulimoviridae*. Our phylogenetic analysis revealed that DNUaV clusters with the monophyletic group K12/T16 (Fig 3A). Since the sequences reported in these previous studies were obtained using a PCR-based approach, the authors were unable to confirm their episomal nature and so theorized that the sequence groups could represent either divergent badnaviruses, ancient endogenous pararetrovirus sequences, or possibly new genera within the family *Caulimoviridae*. The full-length DNUaV sequence presented here provides strong evidence that the sequences in group K12/T16 may also be derived from episomal virus/es infecting yam.

When the yam germplasm collection held at CePaCT was tested for DNUaV using primers designed from DNUaV ORF4, only 2/173 samples tested positive, both of which were *D*. *nummularia* from Samoa. Sequencing of the PCR products from the two accessions revealed 99% nucleotide sequence identity to the full-length RCA-derived sequence, indicating that the sequence was conserved in both isolates. These results suggest that DNUaV does not appear to be integrated into the genome of *Dioscorea* spp. as the only two samples that tested positive with PCR also tested positive using RCA.While this result does not exclude the possibility that DNUaV sequences are either partly or wholly integrated into the genome of the yam species tested, the available evidence suggests the existence of only the episomal form. Sequences with high similarity to DNUaV have previously been identified from *D*. *nummularia* originating from the Solomon Islands [3], however, we were unable to obtain yam samples from the Solomon Islands for testing. The distribution of DNUaV in the Pacific needs to be determined as the current sample set included only two *D*. *nummularia* accessions, both from Samoa.

This research builds on the work carried out previously [3,17] in characterizing caulimoviridae from yams in the Pacific and is important in confirming the episomal nature of reported sequences. An understanding of the episomal virus diversity infecting yam will enable genebanks to test their genetic resources to ensure safe distribution. The diagnostic protocol described here for detecting DNUaV may be suitable for routine diagnostic screening for DNUaV in yam germplasm collections.

## Supporting information

S1 Table Details of yam partial RT/RNase H-coding sequences used in the phylogenetic analysis of DNUaV. (XLSX)

S2 Table Acronyms, GenBank accession numbers and virus names of sequences used for phylogenetic analysis in Fig 3B. (XLSX)

S1 Dataset Complete nucleotide sequence of Dioscorea nummularia-associated virus. (DOCX)
