# Supplementary material for: Characterization of a novel member of the family Caulimoviridae infecting Dioscorea nummularia in the Pacific, which may represent a new genus of dsDNA plant viruses
Source: PLoS One. 2018 Sep 12;13(9):e0203038. doi: 10.1371/journal.pone.0203038 (PMC6135502; doi:10.1371/journal.pone.0203038)
Supplement: S1 Dataset — (DOCX) [file pone.0203038.s003.docx]

Complete nucleotide sequence of Dioscorea nummularia-associated virus

TGGTATCAGAGCAATGGTAGCTTAAACTTCTAAGATCTTCAGTATAAGGGTCTATTCCCTCTTTCTGTAAGACTATAATAATATTTATTATGCTCTGAAAATTTGAAGTATATATGTCTTAATATTCTTTTTCTAAGTTTCAAGGGGAATTTTTGTTTGTTTTTTTATGTCTGTTTTCGATCAGTTGTCTAATTGAGGGGTGAGCTACGGCCAGATCCCTCTTAGAAAACTCTTGTATAGAAGAAGATCCACAGTAGTAGTGGTGACTTCTTCAATGATTTTCTTATTAGAACTACTTAGTAAACTTGCATATTCTTAAAAGATGTTATGCTGGTAAGAGGGGGTAAGCATTTTAAGTAATTAAGCAAAGGAGAATACTTTGATCTGGAAGGTGGAGACTCGATAAGCATCCCTTAAGTGATTTCTAAGTACGCGGAAGGCGGAAAGGATAACTTCAAGGGCTAGTAAGGGCGTAAGACGGTAAGCCTGAAAGATCAAATCAAGTATGTCTCTGATGAAAGAAATGCTTCCTAAAGCTTCTTATTAACATAATAAACTTTGAGAAAAATGTTAGTTATACTATTTAAGTTCTTACTTAGATTATATATTATAATGCTTGTTGCAATTATAAATCTATTACTAACAGTCTATAATATATCTGTTGATATTCATAATATGTGTTATAAAGGTTTGAAAAAACTTTCTGAGCATGCAAATTCTCAGATCGTATTTTTAAACAGAAGGTTTATAGCCCATTAAAAGACAGTCAGTCTTTACACTGTGATTTTGGGAGGCCTGGCCTGGTCCAGTCACTTCTCAAATCCAGAAAGGGAAGACATTGCATAATCTGTCTGGAGGGCAAGAACCGCTGTTTTTCTATGGAAAAATCCCTTGAATATTTAGATCTAGAAGTAAGGCATGTGTATCACAAATTTACTTTGGATAATAAGTATTATTACTGTTTTTCAGAAAAACAAGGGCATAAGCATAGATTCCATACTGAAGATCAACAATTAAATCTTTTGTGTGACTCGTCTTATCATATATTGCATAATCTTACTTGTTTGTGTGAAAAAATTTCAACCAGTATAGCTAAAACTAAAATATCTTTTGAAAAACTTTCAAAAAAATTAGACAATTTAGAAGAAAATCTCTCTTATCTTAAAAATCAGAGAGAAAACAGAAATATACTTATTAAAGAGGAAATTAATCAAATCCTCACAACAGAATTCCAAAAAACTAGACAAAAGAACACAGCTAGTTTTAAATCTTTGGAAGAAAAAATCCAAAGAACAGAATCTTTATTATTAAAACTCATTGGGGAATGAGTACAGAAGATCTAGAGTTAAAATTAACTCTAGAAAAACTTTCAAAAACTGAGTTTACTCCAACTCAAGGATTTACCAAGGAAGTCTCACAGTCTAACTTGATTAGACAACATAATACCATTATTGAACTTCTACTTACTCTACATCAGAAGGTTGATAAATTAACACAAAATGTTAAACAGATCCAAATCAAAGAAGCAGAGCTTAGTTCAGTAGTTAAAAATCTCCAAACGAAAAATAGTACTGAAGAAAAGCCTGTAGTTAAGACATCACCAAAGGTTTTTGCTGTTTGGGAAGACCCTAAACTGCAAGTTGAAGCCTATAAAAGAAAATATGGACAACAAGTCTACAGTCATACTTCCCACAAAGGACATAATTAGGTCCTACAGAAATCAAATTTTTCCTAAAAAGAAAAATAAGGAAATCATTATAGAAAATCTGTTAAATCCTGAACAACAGTTAGATCTGGCACGATCTAAACAAGCAAAATTAATACCAGCAGAGGTTCTCTACCAGCATGGTTTTTGGGAGACAAAACCCAAAGTATACCAACATTATAGTGAAGAGATTATATCTTGTTGTGACACAACAACACAGATAGATTTGAAGTTTTTATCCAAAAATTCAAAACATGAGTTATTATCAAAAGATTATTCCATTATTCACATAGGATTAATCTTGATAGGCATACATGGCCTTCACAGGAGAAATTATGGTTCTAAAGTAATGATAGCATTAGCAGATACTTCTGATAATATACCTCAAAAAGCTATCATAGGATCAATGGAAGTAGACATGGGAGAAAACCACGAGCTATGTTACTTTGCTCCAGATATGTCTATGACCATACAAGATTTTTGTAATTACTTTTCCCTGATTATTAAAACAAAGGGATATGAGACAATGTCTCACAGAGATAATTTACTCATTACAAAAGCCCTAGTAGGAAGAATTGGCAACAATTCTTCTTCAGCATACAGGCTAAAAATTGAAAATGTCCTAACACACCTTGCAACACAAGGAGTTAGGGCAATAGAAGGAAAGAAATTTTCCTCCACAGACTATGATGAAACAGAATGGCAAATCTCACTGAAAGAAAAGAAGAAGATGCCAACAAAATTACAGTATTGGAAAGAGTTGTCAGGCGAAAGCTCAACAGCAACTTTTACATTCACAACACCAGAAGAAAAACCAATCAAAGAGGAAATTAGTGACGAAGAAACATGTTTGTTCTTCATAGATGATTATGATGATGATTGGACATCAGAAGAATTAGAAATTCTCCAAGAAGAATTAGGAGTAATAATTGAAGAACCTTTAAATATTAGAAGAGTTCATACCCCAATTACAGAACAAGTTACAGAAACGATATCTCTTCAGCCTTTGTATGAAGAAGAACCTTTGGTAAACAGAGATATATACCAAAGAATTCTACAAGAGCAAAATCAAGAATTTGAGTGTCCAGGAAGGATGCAACAGTTAGACCAAGTTCTAATGGAAGCAGAATCCGAGAATAGGCCAACAACCTCTGTACCACAAACAAGGGTATCTGGCACACCTGTATTCGGACCACCTGCTCTACAACCAGTGCAACTTCCTCATACTATAATACAACCAAGAGGAAATGTACATACAAGACCGTATAGGTATGATCTTATACCAACTTTACCTTCGGCAAACGTACAAACAGGATCCATGCTAGTTTTACCTGAAATATCTAAATGGGAAGAAACAATTAGAAGATGGGAAAGTAATACCATCACACATGTCGCAACAAAACAATTCACAGACAACCTAGATAAGGTAATGTATGTGGAAAATTTACTAGGAGAGACTGTTAAATTATATTTCCAAAACTGGAGAACAGCTTTTCCTACAGAATTCCAAGCACTAATAGACATAGCTGAAGATACAGCTAACATAACTTCACAAATTCGTCAAATTATATTAGGTTATGACGAATACAGAGGCCAAACAGAAATGCAAAACCAGGCCCTGTTAGATCTAGAACAACTCCAGATCACAAACATGAAAGACATAGAGGCATACTCAACAACATTCTTAGCACTAGCATCTAGGACAGGTGCAGCCTTTCTCAGTCCAGAAATTAGTGCAAAATACTTCAGAAAATTGCCTCCACCTTTCAACATTAGGATTCAGGAAATGTGGACCGAAAAATTTCCAAATTGGCTAGTTGGAGTAGCCCCACGAATTGATTTTACCTACAAAATACTACAAGACCTATGTAGGAACAATGAATTACACAGGCAAGCTAAAGACTTCTCTTTCTGTAGAAGTATAGCAGTGCCAGGCCAATACAGCAGAACTACACAACCCAGAAAAACATTGCGTAGATCTACTAGGTATAAAGGTCACAATGTGAAATCAAACCAAGTTAGGAAGTTTCGAACTGACAAAGCTACAGGTAAACCATCAAAATGTAGGTGTTTTATCTGTGGAGAAATAGGCCATTTTGCTAACCAATGTCAAAACAGAAATGTTAATAGACAACGATTGGCCATATATGAAGAATTGGATCTTGAACCCCAATGGGACATAGTTAGTTTAAATGATGGTGAAGATCCAAATGATTCGGATATATGTTCTTTCTCTGATAATGAGTTAGATCAACAGGAAAGATTAGAATTTCCTACAGAAAAATTTTTAATGATCACTTTAGATTCTATTGATTGGAATCTTCAGAGAGCTAATAAAAAGCTCACAACAGATCAACAAAAATGTATGCACAACTGGAACCATCATCCAGATATTGATTACCTCAATAACAATTGTTATTTTTGCAGATTAAGACCTCCAAAAAGAGCACGAACAATATGTCTTAATTGCAATCTCTTATTATGCCGATATTGTGCAGAGCTAAAATTGAAGATTGAAGTTCCTTTAGGACAACCACCTATCCCTGTTTCTAGAGTGGATCATAGGAGGTTAATCCTAGAACAAGCTCAACATATAACTGAATTAGAACAGGAGTTAGCCCAACAAAAACAAAAAGAAAAAGATTTAGAAGCCCAATTATTAGCAGAAAAACTTTCAGGGTTTCAGTTAGGAGAATCTAGCGAGACAATCCATATGGCTTCAATTGAAGATGAAGAAGGAGTAAATGCTCTTGCTGCAGTTCAGAATAATCTCCTTAATTTTATTGCTATATTAGAAATAGGGGATAAGACTTTCTCCCTTAAAGCAATTCTAGATACGGGCGCATCAGGATGTTGTGTTCAGTATTCGGCCCTTCCAAATTACTGCTATGAGACTTTACCACAACCAGTGAACCTTCATGGTCTCAACAATATTGAAAAAGCCAAAATGAGGATTAGGTCAGGTAACATTATCCTCAACAAAGACAAATATCCTCTGCCTCTAACTTACGTTACTCCTGTTATTTCAGGAGAACTACAGTTAGTCATTGGAATGAATTTCATCCATTCTTTCAAGGGTGGAGTGAAAATAGAAGGAACATCTGTTACCTTCTACAGAAAATCAGACATATTACAAACTTCTCCTATAATAAATAAAAATCTAGTCATAGGAGAAGACGGAGAAGATGTAAACCAAGAAAGTATGTACATGATAGATGACATTCTCCTCTACAATGTAGGAAGGTTAACAGACTACGAGAACCAAATAATACCCATACTACATAAATTGGAAGAAATCCAAATTATTGGGAATGACCCATTAAAGTATTGGGAAAAGAACCAGATTAGATGCAAGCTGGACATCATAAATCCTGATCTTACAATTCAGGACAAGCCTATTATACCTTCACCAGAAATGGCGAAAGAATATGAAAAGCATATTACAGAATTACTGGCACTCAAGGTTATCAGACCTTCCCAAAGTCGCCATAGGACAGCAGCTTTCATTGTCAACAAACATTCTGAACAAGTAAGAGGTAAAAGTCGTATGGTATACAATTATAAGCGACTAAATGATAATACATACAAAGACCAATACACACTCCCAAGTATTGACTATCTCCTACTTAAAATTAAAGACAAGATTGTCTACAGCAAATTTGATCTAAAGTCAGGATTCCACCAGATTATGATGGATCCACAGAGTATAGAATGGACAGCATTTGTCTGCCCACAGGGTCATTTTGAATGGATAGTAATGCCATTCGGACTCAAAAATGCACCTTCAGTATTCCAACGAAAAATGGATAATATATTCAAAAAATATTCAGAATTTGTCTGTGTTTACATAGACGATATTTTGATATTTTCTGAAAGTATACAACAACATGTACAACATTTATTACAGTTCTTCCAAGTATGTAAGGAAGAAGGGCTTATTTTGTCAAAAACAAAATTGAAAATAGGAGTAGCAAATATAGAATTCCTTGGCCTAGAAATAGGAGAAGGAAAAGTTCAACTCCAGCCCCATATATTAAAAAATATATTAGAATTTCCTGAAGACCAATTAGAAACATTAAAGGGTCTACAGAAGTTCTTAGGGATATTAAACTATGCCAGAAATTATATCCCAAATTTGAGTAAATATACTAGAATATTTTATAATAAGTGTTCCAGCAAAGGAGAAAGAAAATTTAATTCTCAAGACTGGAAGATGGTTAGAAAAATCAAAGAAGTAATTACCAAATTACCCCCTTTGATTTTACCAAAACCACAAAGTTATATTATCATAGAGACAGATGGATCTCTAGAAGGATGGGGGGGAATTTTAAAATGGAGACCCTCTCAACAAGATTCACCTTCAACAGAAAAAATTTCACGGTATTGTAGTGGTAGCTATAAATCTGCCATCTCTGCAATAGACGCTGAAATAATGGCTTGTATATATGTATTAGATAAGTTTAAAATATTTTTATATGAGAAAAAAGAATTTACCCTTAGGACAGACTGTATTGCAATTGTAAATTTTTATAACAAACTTAATAATAAAAAATTGAATATAAATCGTTGGGTAAATTTCTGTGATTTAATTACAGGATTAGGTTTAACTGTAAAAATAGAACATATCCAGGGTAAAGATAATGTAGGAGCTGATAGACTCTCTCGAATTATTGCAGCTAGCCCCTACAGATAAAATGGCAGAAAACAAGCCAGCAGACAAAATGACAGAAAATGCAGCCAAAGGGAAAGAGTTGCCACAATTTGTAGAAGGTCAAAAAGGAAATTTCCGAATTCCTTATGGTTTGGAACTAGACAACCAACAGAAAATAATCACAAATGCCCTATGGAGAGCCACCACTTCAACAGCCAAAATAAAAGCATTAAATGCTTTATGCCATTATTTTATAAAAGAAACAAAACAAGATTTTTCTTACTTTGTTATCTTTGAAGGAAAGAAGGCAGGAGTATATTACACATGGGGAAACCTACAGAAAGCTGTAGGCAAAAGAAATACCCCTCAAGGATGGCGTGGGTTTTATACACAACAAGCTGCTGAAACTGCATTCTCTCAATACTCCAAAGCACAGCAAATTATGCCAGACATTCAAAATATTTCTGTCAAAAGAGATGCAGCAGATATTCAATCTGCACAAGATAATAACAAGGAGAAAATCCTAGAAGTTAGTAAAACTTCGAAAAGAAATATGACAGTCTTAGAGGTTGGTGAATCCTCACAGTGTAAGATCAGAATCACAGAGGAAAAAGAAACCTCATCAGAAAAAGATAGATATATACAATTTAATAAATTATCAACCTTATTACACTTGAAAAGATCTTATAGACAAAAATGGTTAGATAATTTACCAAGAGAAGTAAGTCAATTAATAAACAACAAGGTAAAAGAAACATTTTATTTTGACTTACAAATCTCCCAAGAAATTATATATGATCTAGATGAACAGAGAAAGGAAGGATTGGAAGTAGTACAGTGGACCGGGTTGCCAGTACAGAATAGTTTTGCACCAGCTATCTTAATGGCAAAAGTCAACTGGGCTATATTTGAACCAAAGCCATTAGTTGCTGAATTGTTCTTTCATGGTATGCTCTTACTCTTAAGCCTAACAGAAATGGAAGACATACCAGATTTCTTTGGACCCAAATTGCAAAATTTGCTCAGACAGTACAAACGGGATGGAAATTGCGAAATTCATATTATATCTGAGCTACCCAGAATCCGAGGAAATAACATTCTCCCGGCTACTCATCACATACTTCTCAGTAGAACCTTTCCACGATGGCTTAATTATACATCTGGTCCTTTCAACACAAAAAGTATTGGAGACATCTTGTATTATACTGAGTATTTTGTAAATTTTAAAGGACCTTCTGATGATTGGGAATTATTGGGAGAAACTCTCACAGCCAAGGTTTGGGTGCTTCACGGAATAGCATCTAGATGCCGAAGTATCACTAGCATCCCAGGACGTCAAGCTGAAGGATTTGCTATAACTCAGCCAGTAAACCTAACAGATGAAATGGATCTAGAAGGAGCCTCTACAGATTAATATCCTTCTAGAATGGGTAAAAATGTAAAATATGTTATGAATTTTACTTTCCAAAAATACCCTTTGCATAGTAAAAAAGCAAGGGTAAGCTATGTAATCTTATTTCCAGAAAATACCCTTCTATACAGTAAAAGAGAAGGGTAAGGTGTGTAATTTAGAAGGAGGTCTCTCTCTATATAAGAGACCCTCCCTCAGTTGTAAGATATAGAGAAAAAATAATAGAGAATCCTATTCTGAGTTTAATAATCCTTGGCCTCTCTCTTATACTCTGAGTACACAAGGCAAGAAGAAGAAACTCTGAAGTAAGGTATTATCTCAACCTTCGATTCTTTCTTCTCTTCTGTTCTACCTCCTTCTTGTTTTGTAAAC
